# Special Issue: “Molecular Insights into Zoonotic Diseases”

**DOI:** 10.3390/ijms27188339

**Published:** 2026-09-19

**Authors:** Vishwanath Venketaraman

**Affiliations:** College of Osteopathic Medicine of the Pacific, Western University of Health Sciences, Pomona, CA 91766, USA; vvenketaraman@westernu.edu

Infectious diseases continue to cost human lives, causing high levels of morbidity and mortality worldwide (Figure 1).

This Special Issue, titled “Molecular Insights into Zoonotic Diseases”, is dedicated to molecular-level research on host–pathogen interactions, pathogenesis, and the mechanisms of acute and chronic infections, with a focus on zoonotic diseases (Figure 2).

This Special Issue includes six publications that explore the molecular aspects of human-animal interactions and the transmission of bacteria and viruses from animals to humans.

Jurasz et al. (1) analyzed serum and cerebrospinal fluid samples (CSF) from 109 patients with encephalitis for *Torque teno* virus (TTV) infection using serological and molecular testing, virus quantitative measurement, and next-generation sequencing- (NGS)-based phylogenetic analysis. TTV noncoding region (UTR) and/or open reading frame 1 (ORF-1) sequences were detected in the serum of 86 (79%) patients and in the CSF of nine (8%) patients. Five of the latter patients were coinfected with various entero- and herpesviruses. Anti-TTV-IgG was detected in 80 (73.4%) sera samples and in two (1.8%) CSF samples, whereas anti-TTV-IgM was present in three (2.8%) sera samples and none of the CSF samples. Phylogenic analysis of CSF-derived TTV ORF-1 sequences revealed the presence of three unique variants in one patient. TTV was quantified in five CSF–serum pairs: the viral loads were similar in two patients, and the TTV loads were approximately one log higher in three serum samples (1). These results suggest at least occasional TTV replication in the central nervous system (1). However, whether TTV is the cause of encephalitis requires further studies.

Viral metagenomic next-generation sequencing (vmNGS) has transformed the untargeted detection and characterization of (re)emerging zoonotic viruses, surpassing the limitations of traditional targeted diagnostics. In their review, Russel et al. critically evaluated the current landscape of vmNGS, highlighting its integration within the One Health paradigm and its application to the surveillance and discovery of (re)emerging viruses at the human–animal–environment interface (2). This review provides a detailed overview of vmNGS workflows including sample selection, nucleic acid extraction, host depletion, virus enrichment, sequencing platforms, and bioinformatic pipelines, all tailored to maximize sensitivity and specificity for diverse sample types (2). Through selected case studies, including SARS-CoV-2, mpox, Zika virus, and a novel henipavirus, Russel et al. illustrate the impact of vmNGS in outbreak detection, genomic surveillance, molecular epidemiology, and the development of diagnostics and vaccines (2). The authors further examined the relative strengths and limitations of vmNGS in passive and active surveillance, addressing barriers such as cost, infrastructure requirements, and the need for interdisciplinary collaboration (2). By integrating molecular, ecological, and public health perspectives, vmNGS is a central tool for early warning, comprehensive monitoring, and informed intervention against (re)emerging viral threats, underscoring its critical role in global pandemic preparedness and zoonotic disease control (2).

Cruz et al. (3) reviewed the pathogenesis of zoonotic pathogens *Mycobacterium marinum* (*M. marinum*) and *Mycobacterium leprae* (*M. leprae*) through the role of reactive oxygen species (ROS) in redox imbalance, immunity, and cutaneous wound healing. Physiological ROS levels are vital for T-cell activation and differentiation. However, excessive ROS production, particularly in innate immune cells, can lead to T-cell suppression. *M. leprae* infection is associated with significant reductions in the levels of key antioxidants such as glutathione (GSH), GSH peroxidase (GSH-Px), and GSH reductase (GR), reductions that correlate with disease severity. For *M. marinum*, disrupting the pathogen’s redox balance through thioredoxin reductase (TrxR) inhibition sensitizes bacteria to ROS damage, reducing bacterial load. Overall, redox imbalance is central to the pathogenesis and persistence of cutaneous mycobacterial infections, compromising host defense and impairing tissue repair. Restoring and maintaining proper redox homeostasis, potentially through the role of GSH as an antioxidant, represents a promising adjunct treatment to improve host outcomes in these challenging dermatological conditions (3).

The One Health concept recognizes the close interconnection between human, animal, and environmental health (Figure 3).

In recent years, the One Health perspective has intensified the scientific focus on zoonoses. Among these, arboviruses—viruses transmitted by arthropod vectors—represent an emerging challenge, particularly in the present period, which is strongly conditioned by climate change. Laratta et al. (4) contributed a review on *Usutu virus* (USUV) to this Special Issue. USUV is a *Flavivirus* maintained via an enzootic bird–mosquito–bird cycle that infects other vertebrates. USUV is currently a serious animal health concern due to its expanding host range and increasing avian mortality events. Although USUV appears to be less dangerous to humans than other emerging arboviruses, the neurological disorders caused by USUV are alarming and increase the need for a better understanding of the spread and genetic evolution of USUV, as well as for the stronger promotion of vaccine and antiviral development (4). As with other arboviruses, treatment for USUV is limited to avoiding contact with mosquitoes, which is not always possible. Because vaccines do not yet exist for USUV, the use of modern OMICS sciences may provide comprehensive knowledge for developing effective control and prevention measures to avoid future pandemics and contain current epidemics (4).

This Special Issue also includes a review on porcine cytomegalovirus/porcine roseolovirus (PCMV/PRV) by Denner (5). PCMV/PRV is a porcine herpesvirus that significantly reduces the survival time of porcine xenotransplants in nonhuman primates. The virus was detected in all the examined organs of baboons transplanted with PCMV/PRV-positive organs, and PCMV/PRV was transmitted to the first human recipient of a pig heart, contributing to the patient’s death. PCMV/PRV induces consumptive coagulopathy and thrombocytopenia in xenotransplant recipients. Initial studies in baboons revealed that the virus increased the release of tumor necrosis factor α (TNFα) and interleukin 6 (IL-6), as well as elevated the levels of tissue plasminogen activator (tPA) and plasminogen activator inhibitor 1 (PAI-1) complexes (5). Because no evidence exists that PCMV/PRV infects primate cells, including human cells, the virus appears to directly interact with immune and endothelial cells, disrupting cytokine signaling and coagulation pathways. The highest viral load was detected in the explanted pig heart, suggesting active replication at this site (4). Additionally, cells expressing PCMV/PRV proteins were identified in all the examined baboon organs, where pig cells were also found. Because PCMV/PRV affects only xenotransplant recipients and not healthy humans, this condition should be classified as a xenozoonosis (4). Antibodies against human herpesvirus 6 (HHV-6) cross-react with PCMV/PRV and may contribute to protection against infection in humans. Further research is needed to uncover the molecular mechanisms underlying this xenozoonotic disease (5).

Antiretroviral therapy (ART) has significantly improved the prognosis for those with human immunodeficiency virus type 1 (HIV-1) infection. Although ART can suppress plasma viremia below detectable levels, ART cannot eradicate the HIV-1 DNA (provirus) integrated into the host cell genome. This integration often results in unrepaired DNA damage due to the HIV-1-induced inhibition of DNA repair pathways. Furthermore, HIV-1 infection causes telomere attrition in host chromosomes, a critical factor contributing to CD4+ T-cell senescence and apoptosis. HIV-1 proteins can induce DNA damage, block DNA replication, and activate DNA damage responses across various organs. In a review, Tolomeo et al. (6) explored multiple aspects of the intricate interactions between HIV-1 and the host genome involved in CD4+ T-cell depletion, inflammaging, the clonal expansion of infected cells in long-term-treated patients, and viral latency. The authors discuss the molecular mechanisms of DNA damage that contribute to comorbidities in people with HIV-1- and highlights emerging therapeutic strategies targeting the integrated HIV-1 provirus.

## Figures and Tables

**Figure 1:**
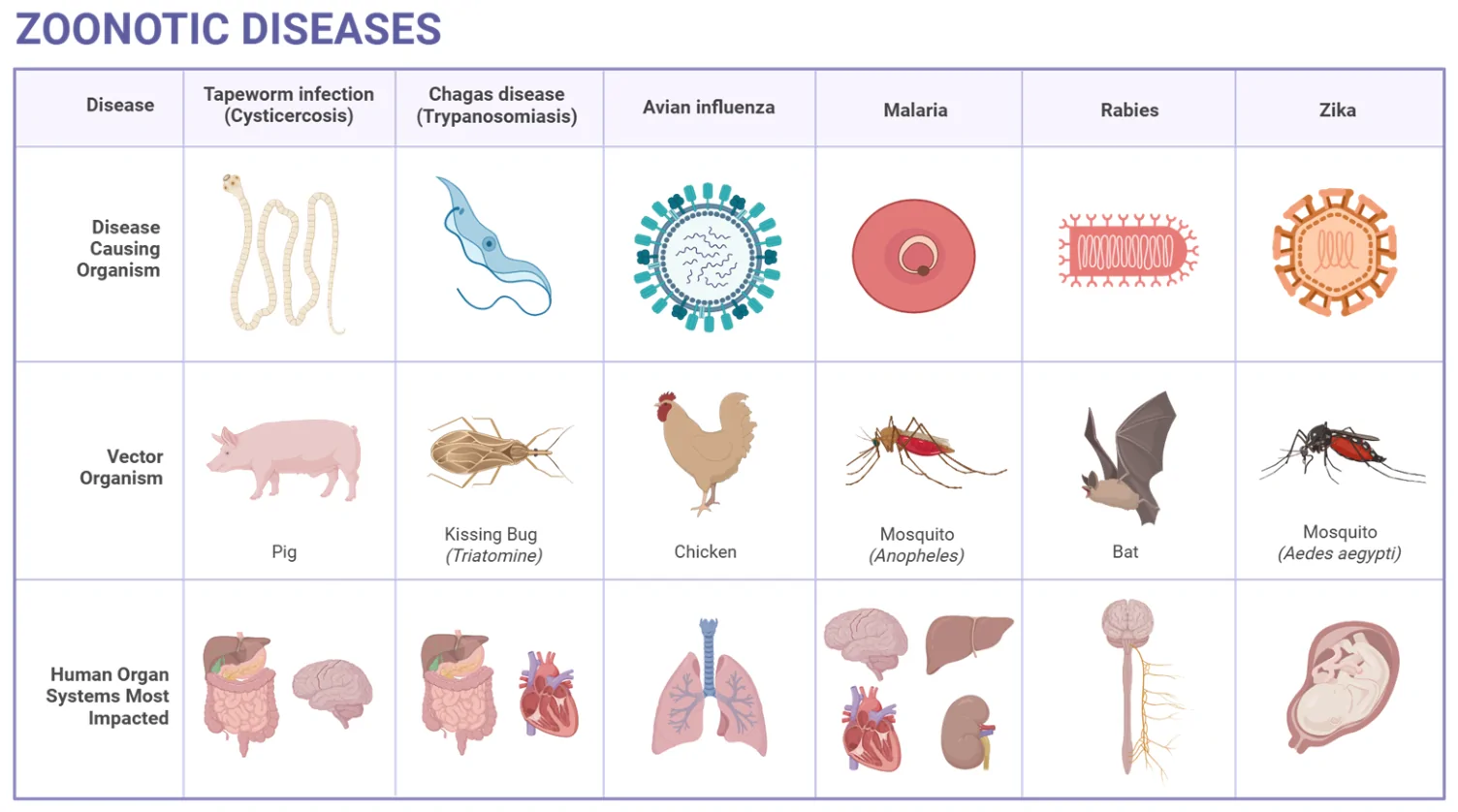
Infectious diseases and impact on human organ systems.

**Figure 2:**
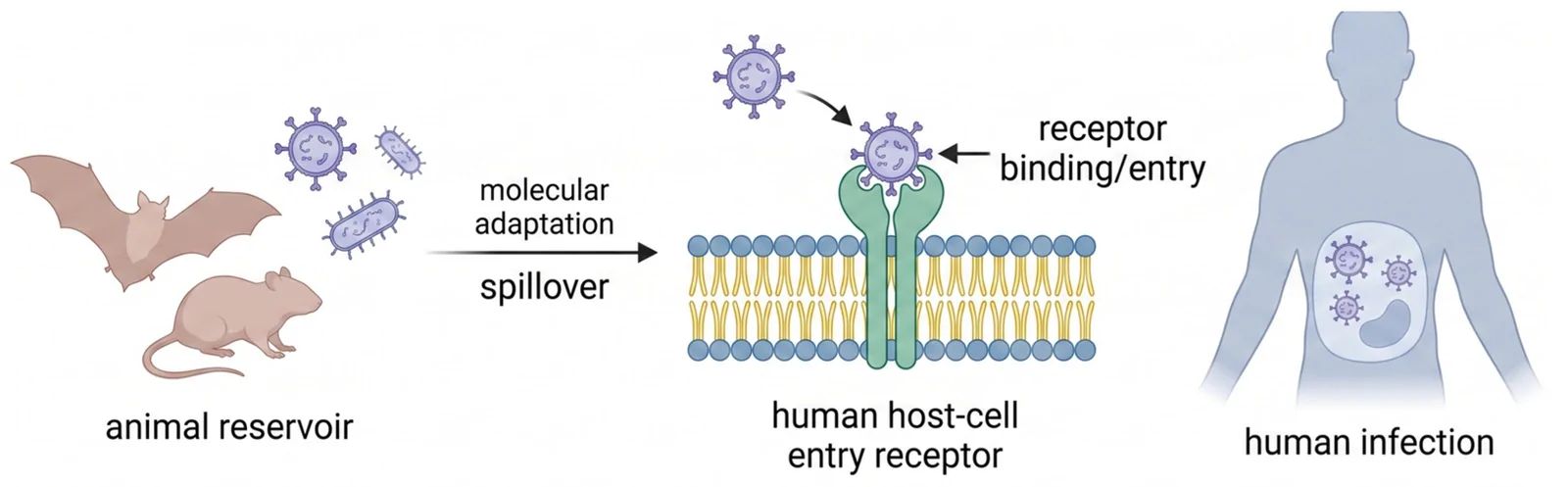
Host pathogen interactions and molecular pathogenesis.

**Figure 3:**
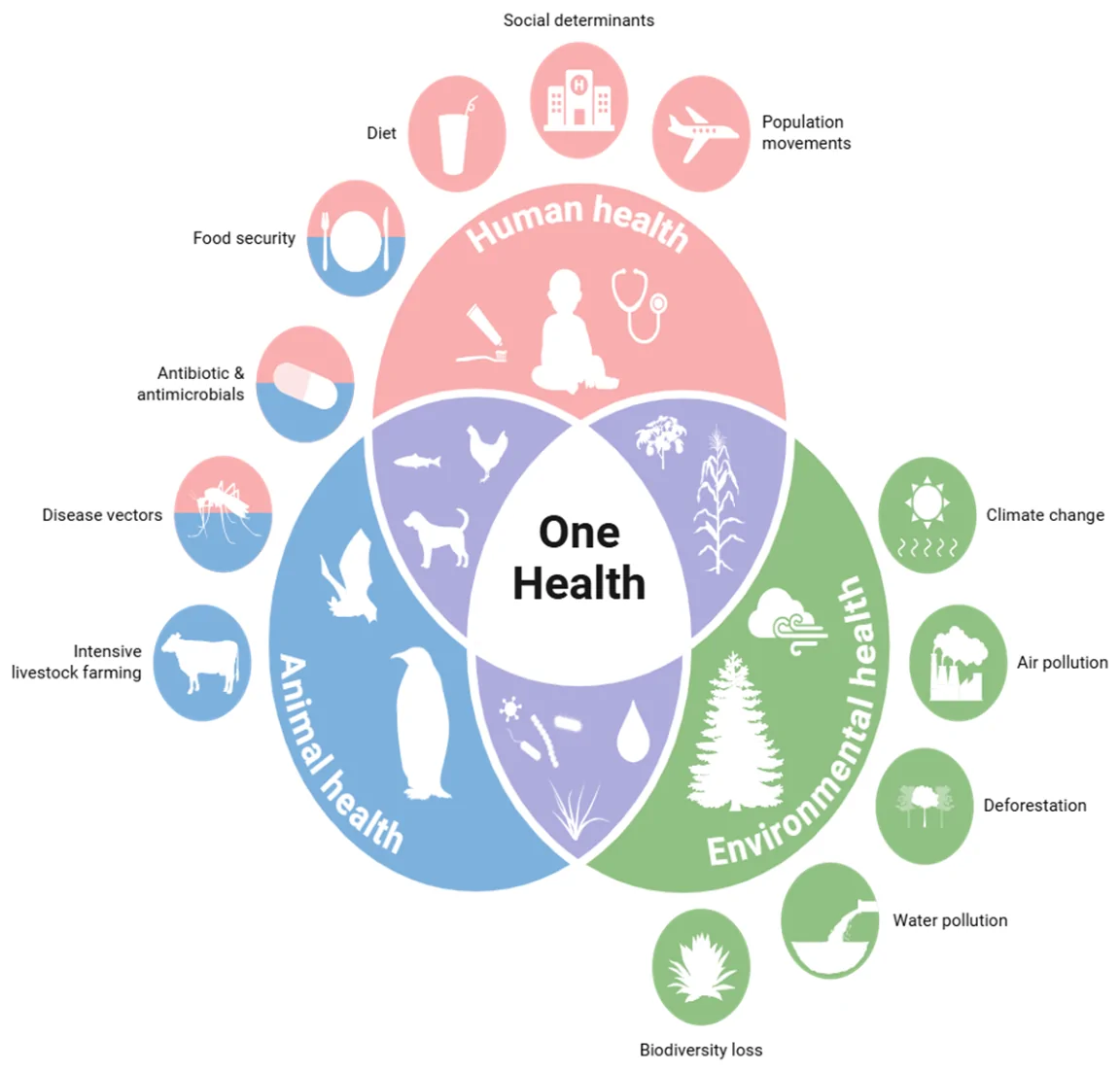
One Health concept.

